# Squalene Peroxidation and Biophysical Parameters in Acne-Prone Skin: A Pilot “In Vivo” Study

**DOI:** 10.3390/ph16121704

**Published:** 2023-12-08

**Authors:** Giorgia Condrò, Roberta Sciortino, Paola Perugini

**Affiliations:** 1 Department of Drug Sciences, University of Pavia, Via Taramelli 12, 27100 Pavia, Italy; giorgia.condro01@universitadipavia.it (G.C.); roberta.sciortino01@universitadipavia.it (R.S.); 2 Etichub, Academic Spin-Off, University of Pavia, Via Taramelli 12, 27100 Pavia, Italy

**Keywords:** acne vulgaris, squalene, peroxidation, in vivo evaluation

## Abstract

Nowadays, acne vulgaris therapies are often unsuccessful. One of the responsible factors for the formation of comedones and inflammatory lesions could be the peroxidation of squalene, a hydrocarbon representing one of the major components of human sebum. This peroxidation is increased by solar irradiation. The purpose of this work was to set up an in vivo method for the extraction and quantification of squalene from acne skin and to correlate the results with biophysical skin parameters such as sebum amount, protein content and TEWL. Healthy volunteers were used as control. The results obtained demonstrated that acne-prone skin had a major quantity of squalene, and, in the stratum corneum area, its peroxide form is present. Moreover, Spearman’s rank correlation showed a positive correlation between sebum content and peroxide squalene and between porphyrin intensity and peroxide squalene.

## 1. Introduction

Currently, acne vulgaris represents one of the most prevalent chronic inflammatory diseases affecting not only adolescents but also adults [1]. Different features of acne patients are seborrhea, inflammatory and non-inflammatory lesions. They mainly occur in sebaceous areas such as face and upper trunk [2]. Among them, sebum production and its alteration are aspects that need to be taken into consideration [3]. Basically, in healthy people, sebum is formed by several lipids such as triglycerides, wax esters, squalene, free fatty acids and a smaller amount of cholesterol, cholesterol esters and diglycerides [4]. These epidermal surface lipids contribute to normal skin functions such as barrier protection and skin maintenance. One of the main components is squalene, an unsaturated hydrocarbon precursor of steroids, consisting of six isoprene units [5]. The literature shows that squalene is present in a higher percentage in acne vulgaris [6].

Moreover, due to its unsaturated structure, it would undergo a peroxidation process that would worsen the pathological condition. In fact, the peroxides appear to worsen the acne lesions [7]. Chemically, squalene is a highly susceptible molecule to oxidative phenomena due to the presence of six carbonic double bonds that bind atmospheric oxygen. When squalene is oxidized, it generates six isomers of squalene monohydroperoxide. From a dermatological point of view, oxidized squalene is irritating and strongly comedogenic.

Squalene oxidation appears to be a process that can produce microaerophilic conditions, and thus create an environment suitable for bacterial colonization [8].

Squalane instead is the result of the hydrogenation process of squalene. It is the fully saturated form, which means it is not subject to autoxidation. Small amounts of squalane are found in sebaceous secretions, so it is a natural product [9].

**Figure 1:**
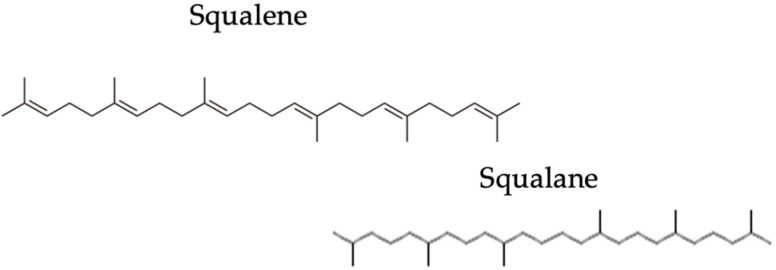
Chemical differences between squalene and squalane.

Figure 1 illustrates the chemical differences between squalene and squalane. As mentioned earlier, the squalane represent the saturated form of squalene.

**Figure 2:**
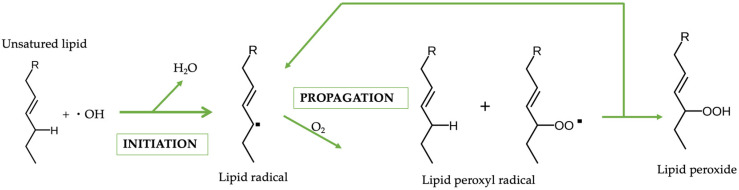
Mechanism of lipid autoxidation reaction.

Squalene peroxidation belongs to the lipid autoxidation reaction. This process consists of two steps: initiation and propagation. Specifically, when the compound involved in the reaction is a lipid, the process is called peroxidation. Lipids are the main targets of free radical attack and, therefore, lipid peroxidation has a profound impact on the biology of living systems. The initiator of the process is a hydroxyl radical; it is self-propagating and susceptibility to oxidation increases as lipids become unsaturated. In the second phase of propagation, the free radicals formed are very reactive and unstable, so they tend to steal electrons from other contiguous molecules that will undergo the same mechanism. For this reason, it is a self-propagating reaction (Figure 2) [10,11].

Several factors contribute to peroxidation process: porphyrins, lipases, metalloproteases secreted by *Cutibacterium acnes* but also UVA rays and cigarette smoke [12]. The amount of porphyrins present is closely dependent on the *Cutibacterium acnes* phylotype that governs the skin microbiota. *C. acnes* type I strains produce significantly more porphyrins than other phylotypes. Type II strains instead produce low levels of porphyrins; in fact, they are associated with healthy skin, and they carry a gene, deoR, a repressor element of porphyrin biosynthesis [13]. The main porphyrin identified in patients with acne is coproporphyrin III, which is present in large amounts in acne lesions, and it is responsible for the inflammatory process [14]. In addition, solar radiation exposure can aggravate skin condition [15]. Squalene peroxide can induce an inflammatory response of keratinocytes through the activation of LOX, a lipoxygenase enzyme capable of producing conjugated hydroperoxides through the oxidation of polyunsaturated fatty acids. Moreover, it increases the production of proinflammatory cytokines IL-6. M. Ottaviani et al. investigated the possible role of oxidized squalene in the development of inflammatory events. For this purpose, an HaCaT human keratinocyte cell line was treated with oxidized squalene. The results showed that squalene was able to stimulate cell proliferation and LOX activity. In addition, an enhancement of NF-kB, a protein complex functioning as a transcription factor that regulates the immune response to infection, was noted, followed by an increase in the expression and secretion of the proinflammatory cytokine IL-6 and triggering a hyperproliferative response of sebaceous duct keratinocytes. Thus, peroxide squalene plays an important role in the pathogenesis of acne by exerting pro-inflammatory activity on the pilosebaceous unit [7]. Squalene appears to be upregulated compared to the rest of the lipids. This molecule, therefore, could serve as a lipid marker for acne-prone skin. The preferred method in the literature for the quantification of squalene from peroxide squalene is high-performance liquid chromatography (HPLC).

Although there are guidelines for the management of acne vulgaris, research and knowledge gaps are still present, especially regarding the assessment of tools able to better help the characterization of acne vulgaris and the measurements of outcomes for assessing acne treatments [16].

The main objective of this work was to set up an “in vivo” test, in which it was possible to analyze simultaneously, on the same area, several parameters. In particular, the biophysical skin parameters such as sebum amount, protein content, and TEWL. The presence and the amount of squalene and its peroxide form were evaluated in subjects with acne-prone skin.

For this purpose, a preliminary in vitro phase to investigate the formation and number of peroxides into squalene over time after exposure to ultraviolet radiation was set up. After that, the in vivo study was carried out on acne subjects (with inflamed and non-inflamed areas) and on healthy volunteers. All participants were chosen by a specific questionnaire based on exclusion and inclusion criteria to obtain a homogeneous panel. Then the subjects’ sebum was collected by the tape stripping method, squalene was extracted and quantified with an HPLC validated method. Furthermore, the biophysical parameters such as pH, amount of sebum, TEWL, and protein content between acne and healthy subjects were also investigated.

## 2. Results

### 2.1. In Vitro Peroxidation Analysis

**Figure 3:**
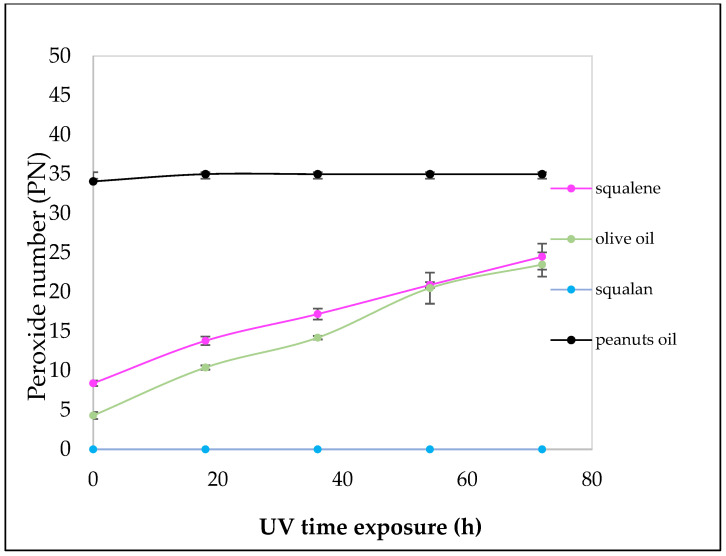
Peroxidation kinetics in solar simulator of the samples. The squalane was used as negative control, while fresh olive oil was used as positive control.

To quantify the peroxide number (PN) obtained, sodium thiosulphate titration method was used. The analyses are conducted in triplicate and are expressed as the mean *±* S.D. at different time intervals until plateau is reached. At 72 h in solar simulator, all the samples reach the plateau: fresh olive oil reached 23.5 meq O_2_/Kg *±* 1.55, peanut oil 35 meq O_2_/Kg *±* 0.43, squalene 24.5 *±* 1.67. All the samples analyzed reach the plateau of the PN value after 72 h of exposure to solar light. Squalene exhibits a low PN at t0 increasing overtime until it reaches 25 meq O_2_/Kg at the end of the experiment (Figure 3).

Natural oils are considered fresh when the NP is <7. In fact, at time 0, the NP of fresh olive oil is about 4.3 and reaches a value of 23.5 meq O_2_/Kg after 72 h of solar exposure. Peanut seed oil showed a very different behavior; indeed, the PN value was 35 meq O_2_/Kg at the beginning of the study. Squalane, used as a negative control, cannot undergo peroxidation. In fact, after the solar exposition, the titration confirmed that no peroxides were present.

### 2.2. HPLC Analysis

The calibration curve obtained for the quantification of squalene had a concentration range of 5 to 200 µg/mL. In Table 1, results of regression analyses are reported. The detection limit (LOD) and the quantification limit (LOQ) were calculated from the analytical curves that are shown in Table 2.

The triplicate of each calibration curve was made in three days, and they showed R^2^ close to 1, indicating an excellent linearity (see Table S2 supplementary material).

Next, the analysis of ANOVA for linearity was determined. The value obtained for the F calculated was lower (0.0040) than the F tabulated (3.467).

There was no significant difference between the values obtained from the calibration curves (*p* = 0.996) (details at Table S3 supplementary material).

For a proper quantitative analysis in HPLC, it is fundamental to assess the precision of the analyte evaluating the differences between the results obtained for the same concentration. The RSD% was found to be less than 5.0%. Therefore, we can state that the method is precise (Table S4, Supplementary Material).

Accuracy based on studies of analyte recovery from samples was made by adding three different percentages of SQ in the samples. The average recovery ranged from 99.10 to 106.51%. The results accuracy was also less than 5.0%. Therefore, the chromatographic method has some accuracy.

### 2.3. In Vivo Experiments

#### 2.3.1. Collection of Sebum

The amount of sebum taken out through the adhesive discs is approximately 600–500 µg for the first strip, 500–400 for the second strip and in the end 300–250 µg. The amount taken is sufficient for the squalene quantification. For the first strip the mean amount of sebum collected (µg/strip) is 700 ± 141.42; for the second strip the value is 450 *±* 70.71, and for the third strip 290.00 ± 14.14.

#### 2.3.2. Squalene Quantification in the “In Vivo” Study

**Figure 4:**
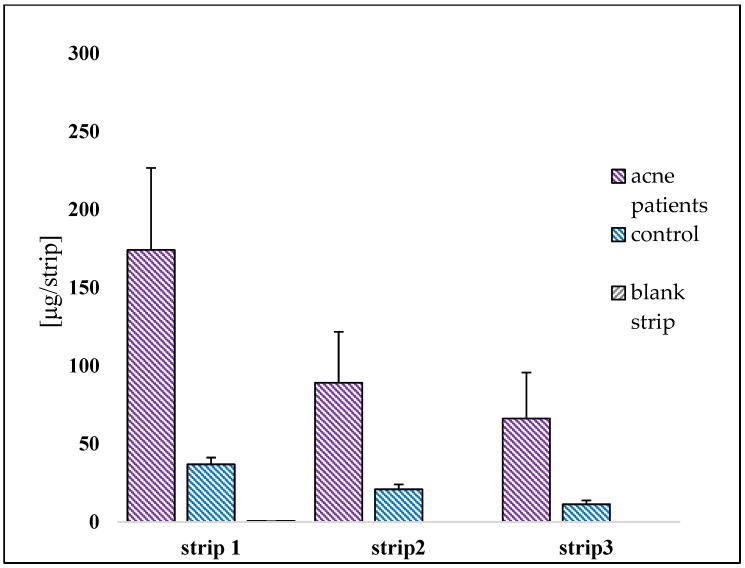
Amount of squalene collected (µg/strip) in the three strips collected on the face. The results are expressed as mean ± S.D.

The amount of squalene both in acneic and healthy volunteers was measured by HPLC analysis. Figure 4 shows a general overview of the results expressed as mean ± S.D./strip. Comparing them, there is a substantial difference between the two groups. In acne-prone skin, the average concentration is 174.124 ± 54.96 μg/strip compared to 36.97 ± 4.55 μg/strip for healthy skin. Acneic subjects show a higher amount of squalene than healthy volunteers in all three strips. The percentage of variation between acne skin and healthy skin is 78.80% for the first strip, 76.53% in the second strip and 82.88% in the third strip.

The blank strip gives no interference either for the extraction method or for the quantification of squalene using HPLC method.

**Figure 5:**
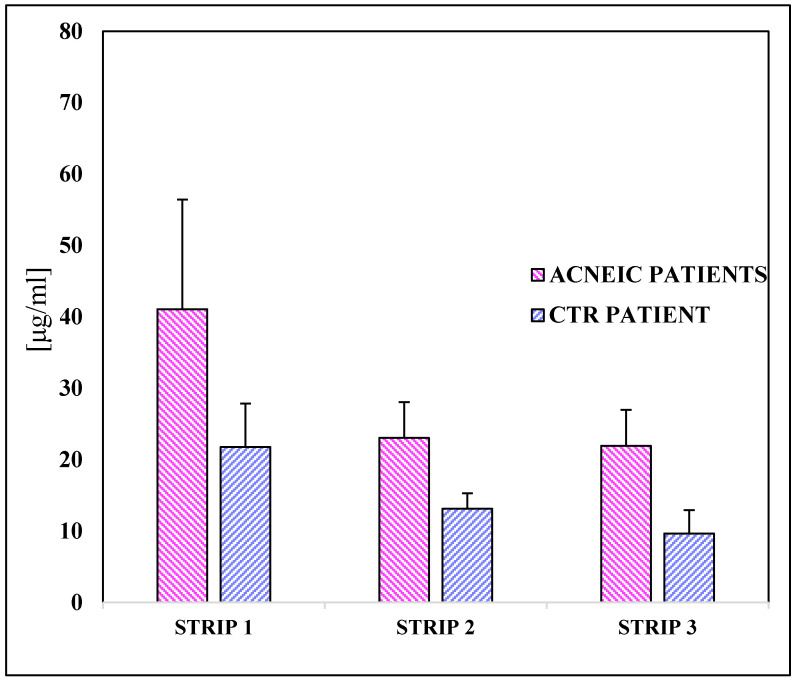
Amount of squalene collected (µg/strip) in three strips in the shoulder area. The results are expressed as mean ± S.D.

The presence of squalene was also compared analyzing the shoulder area of acneic and healthy subjects (Figure 5). In this case, the amount of recovered squalene was lower than those recovered in the face but, regardless, the quantity of the compound was more widely detected in the acneic people. The percentage of variation is lower than the previous results: 47.04% in the first strip, 43.09% in the second strip and 56.06% in the third strip.

**Figure 6:**
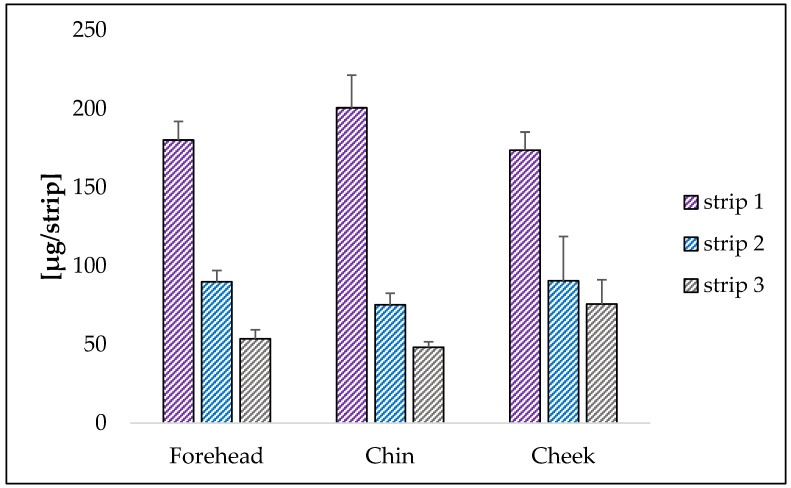
Amount of squalene (µg/strip) in the three strips collected in several inflamed areas of the face. The results are expressed as mean ± S.D. Ten subjects were taken into consideration.

The test provided the quantification of squalene by sampling three different inflamed areas in acneic patients. For all investigated zones, a greater amount of squalene is again found in the first strip reaching a concentration of 180.05 µg/strip in the forehead, 200.63 µg/strip in the chin and 173.64 µg/strip in the cheeks. The value is practically the same independently from the zone (Figure 6).

The amount of squalene decreases in the second and third strips to 89.88 µg/strip and 53.58 µg/strip for the forehead, 75.16 µg/strip and 48.20 µg/strip for the chin and 90.46 µg/strip and 75.69 µg/strip for the cheeks.

#### 2.3.3. Example of a Chromatogram of a Healthy and an Acneic Volunteer

**Figure 7:**
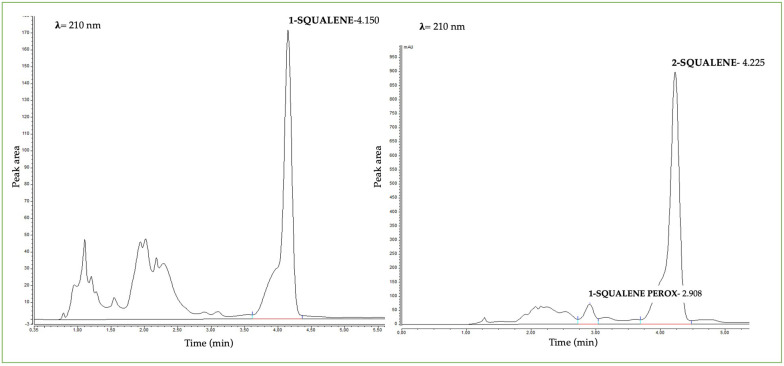
Chromatogram examples of the presence of peroxide squalene in an acneic subject (**right side**) compared to a subject with healthy skin (**left side**). The results are reported as peak area on the y axis, while time (in minutes) on the x axis. The wavelength used to analyze samples was 210 nm.

The presence of squalene and its peroxide form in HPLC is detected through a different time of retention. Figure 7 shows an example of the presence of peroxide squalene in acneic subject (right side) compared to a subject with healthy skin (left side). The presence of peroxide squalene was revealed at 2.908 min. Instead, the squalene in the samples has little difference in retention time, probably due to the complexity of the matrix skin.

#### 2.3.4. Quantification of Peroxide Squalene

**Figure 8:**
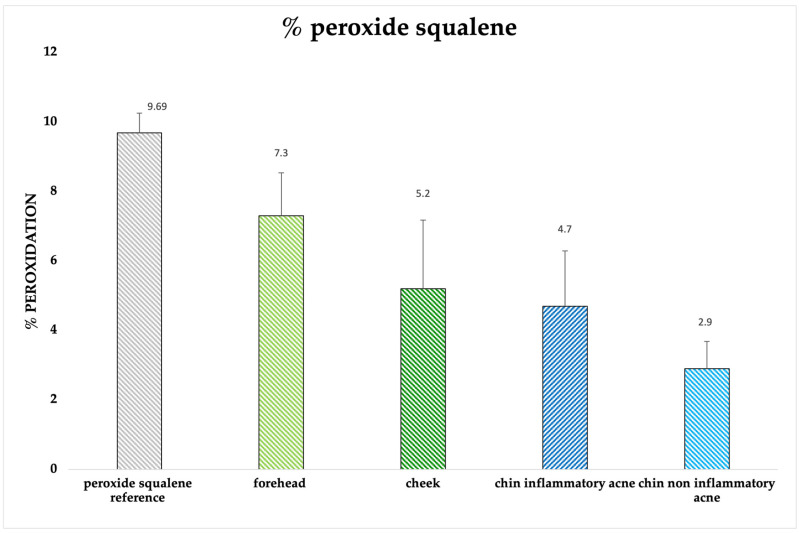
Percentage of peroxide squalene found on samples coming from acneic skin, using peroxide squalene as control. Samples were taken from forehead, cheek and two different areas of the chin (inflammatory and not inflammatory zone). Ten acneic patients were analyzed.

The presence of peroxide squalene was investigated by analyzing different acne-prone face areas: forehead, cheeks and both inflamed and non-inflamed chin. (Figure 8). Forehead shows a higher % of peroxide squalene (7.3%) than the other areas compared to 4.7% found in the inflammatory chin and 2.9% in the non-inflammatory chin. The % peroxide squalene in the cheek shows to have an intermediate value of 5.2%. The analyses were compared with squalene peroxide subjected to chemical oxidation and after solar exposure, obtaining a peroxidation of 9.69%. However, no peroxide form was found in healthy volunteers.

#### 2.3.5. Skin Biophysical Parameters Acquisition

For the acquisition of biophysical skin parameters, a series of probes were used without carrying any pain and damage to the skin and to the patient. Results concerning instrumental analysis of skin parameters are expressed as a mean ± S.D. (Table 2).

**Figure 9:**
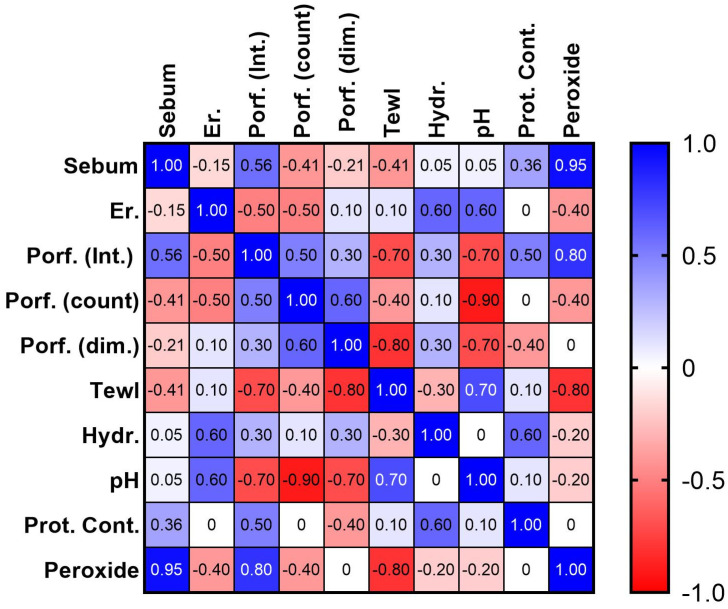
Spearman’s rank correlation coefficients obtained from parameters collected on acne-prone skin. Er = Erythema; Porf. = porphyrin; Int = intensity; dim = dimension; Hydr = hydration; Prot. Cont. = protein content; Peroxide = amount of peroxide squalene.

Figure 9 shows a statistical correlation among all parameters collected in acne-prone skin. A positive correlation was found between sebum content and porphyrin intensity, between sebum content and peroxide squalene, and between porphyrin intensity and peroxide squalene. A negative correlation was found instead between porphyrin and erythema, porphyrin and TEWL and between porphyrin intensity and skin pH.

## 3. Discussion

Acne vulgaris is one of the major skin diseases affecting both adolescents and adults. Its onset may depend on several factors, and the outcome occurs with the formation of inflammatory and non-inflammatory lesions, presence of comedones and alterations in sebum composition. In the present work, we focus our attention on the alteration of one of the main components of sebum: squalene. In particular, the aim was to detect the presence of squalene and its peroxide form in acne-prone skin compared to healthy skin. Moreover, the obtained results have been correlated to some biophysical parameters. The choice to investigate this aspect relates to an absence in the literature of a standardized in vivo method aimed to evaluate the peroxide and non-peroxide form. Furthermore, correlations to biophysical skin parameters in acneic skin have not been reported in the literature.

The work was divided into several steps: first, the selection of subjects has been a key point to obtaining a homogeneous panel. Indeed, men, menopausal women, adults with chronic diseases and women undergoing hormone therapy were excluded. Then, the collection of sebum and thus the squalene was performed with a tape stripping method, validated in a previously recent publication [17]. The purpose was to collect enough material useful for the extraction and the quantification of squalene compound. Different areas were investigated: forehead, chin, and cheeks. Moreover, the shoulder was used as internal control of the same subject, while volunteers with healthy skin were compared to those with acne.

To extract squalene, different methods were reported in the literature. Ayano Ishikawa and Shimizu N. extracted it using cotton swabs soaked in acetone and then extracted in chloroform [18,19]. Swarna Ekanayake Mudiyanselage, instead, used Sebutapes ^®^ to collect sebum on volunteers soaked in 1 mL of ethanol solution 70% [20]. To avoid the use of these solvents, an alternative method is proposed in this study. Both ethanol and acetonitrile were used as extraction solvents. However, the ethanol procedure was more efficient than acetonitrile as an extraction solvent.

To quantify the amount of squalene, a modified HPLC method was used after long research in the literature. For instance, Ekanayake Mudiyanselage S. et al. quantify the amount of squalene with a mobile phase made of 1:1 ethanol and methanol [20]. Again, Chiba K. et al. [21] used a mobile phase consisted of 100% of methanol with a flow rate of 2.5 mL/min. At the end, Bavisetty S.C. and Narayan B. used a 100% Acetonitrile as an eluent solvent [22]. After several experiments, our method consisted of a mobile phase of 70% ethanol and 30% acetonitrile.

To simulate the peroxidation of squalene induced by porphyrins, in vitro squalene was placed under strongly oxidizing conditions, in the presence of 10.8% hydrogen peroxide and in solar simulator for 72 h. After analyzing the solution in HPLC-UV-VIS, a peak was identified at 2.9 min related to peroxide squalene.

Moreover, the formation of peroxides was induced through solar simulator at different times with olive oil (positive control), peanut seed oil, and squalene, and squalane used as negative control. An increase of NP was found proportional to the increase in exposure time.

Observing the results, the tape stripping method allows for the collection a homogeneous amount of squalene/strip. In fact, despite the skin type (acneic and non-acneic) the test was efficient to extract and quantify sebum, and thus the squalene. Since the first strip represents the superficial portion of the stratum corneum, the amount of squalene collected is present in the greatest quantity.

The method applied appears to be successful to also quantify the peroxide form of squalene in the skin. In fact, it was found only in acneic subjects in the first strip, according also to the literature, where it has been stated that several factors such as the solar radiation exposure increases the formation of reactive oxygen species in the lipid skin surface, leading also to the formation of peroxide squalene [23,24].

Therefore, this method could be a good opportunity, in the future, to evaluate both peroxide squalene and microbiota composition.

Simultaneously to the evaluation of squalene, the method used was also efficient for the analysis of the health of the skin using painful probes for the evaluation of biophysical parameters: pH, TEWL, protein content, sebum level. From the results obtained, TEWL has been revealed to be higher in acneic people than to healthy people. This is probably due to the heterogeneity of the structure of the stratum corneum and of corneocytes. In addition, the amount of protein content is higher in acneic subjects, in which the skin barrier is surely compromised. Another parameter is sebum levels, which are higher in acne-prone skin than healthy skin. This aspect correlates to one of the features of acne vulgaris—seborrhea—and it also confirm the presence of a major quantity of squalene in the first group.

The results obtained by statistical analysis are very interesting, showing a strong correlation between porphyrin or sebum content and peroxide squalene found in acne-prone skin. These results suggested that further in vivo studies carried out on many subjects are required.

## 4. Materials and Methods

### 4.1. Materials

The materials used were: D-100-D-Squame Stripping Discs on a polyester carrier sheet (22.0 mm ∅) (D-Squame^®^, Clinical and Derm, 12221 Merit Dr Ste 940 Dallas, TX 75251, USA); ethanol absolute HPLC grade; acetonitrile HPLC grade; PTFE syringe filter 25 mm 0.22 µm (Sharlab, S.L., Barcelona, Spain), sterile Eppendorf tube 2 mL (Eppendorf, S.r.l, Milan, Italy); starch salt 1% (Titolchimica S.p.A., Rome, Italy); ultra-pure potassium iodide 99% (Titolochimica S.p.A., Rome, Italy); sodium thiosulfate (Titolchimica S.p.A., Rome, Italy); glacial acetic acid (Carlo Erba reagents S.r.l, Milan, Italy); chloroform (Carlo Erba reagents S.r.l, Milan, Italy); hydrogen peroxide (Industria Farmaceutica NOVA ARGENTIA S.p.A, Gorgonzola, Milan, Italy); squalene (Sophim, Peyruis, France); peanut oil (Esselunga market, Pavia, Italy); olive oil (own production); and squalane (Sophim, Peyruis, France).

### 4.2. Methods

#### 4.2.1. In Vivo Experiment

The in vivo study was carried out according to the Helsinki declaration (Ethical Principles for Medical Research Involving Human Subjects) [25]. The studies were performed on 10 female volunteers with acne vulgaris and five with healthy skin aged 20–30, chosen based on the selected inclusion and exclusion criteria. Subjects were treated after their personal informed consent was obtained according to Italian law (GDPR 2016/679).

All measurements were made in an air-conditioned room with a controlled temperature and humidity (T = 22 °C, relative humidity [RH] = 70%).

#### 4.2.2. Selection of Acne Patients

**Figure 10:**
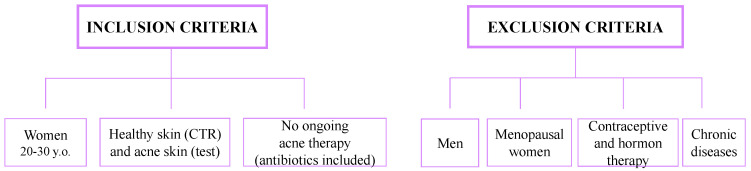
Flowchart of inclusion and exclusion criteria of acne patients.

The selection of acne patients was scrupulously achieved by filling out a questionnaire based on 74 questions with the aim of having a complete history and background of each subject. In particular, the main inclusion and exclusion criteria are shown in Figure 10. Specifically, women who were not on hormone and/or antibiotic therapy and had no chronic diseases were recruited.

#### 4.2.3. Acquisition of Biophysical Parameters

The instruments used for the evaluation of skin parameters involved contact between the skin and a series of probes that did not cause pain or damage to the skin.

The chemical and physical characteristics of various skin sites, such as pH, temperature, and humidity, have an important impact on the composition of the skin microflora. The parameters chosen were pH, total quantity of sebum, erythema, hydration of the superficial stratum corneum, trans epidermal water loss (TEWL) and the amount of keratin protein in the stratum corneum (SC).

Skin parameters were evaluated using an MPA 580 multiprobe adapter system cutometer (Courage & Khazaka electronic GmbH, Cologne, Germany).

TEWL measures the amount of water lost through the skin in the form of water vapor and varies depending on the hydration status of the skin. TEWL reflects the integrity of the skin barrier and, therefore, is used as a benchmark to assess skin health [26,27,28]. Low skin hydration results in trans epidermal water loss with altered barrier function. For this reason, the measurement of TEWL (Trans epidermal Water Loss) could be used to understand the status of the skin barrier, and consequently also gain insight into the composition of the skin’s bacterial flora.

A TM 300 Tewameter probe was used to measure this parameter. The amount of TEWL is expressed in g/h/m^2^. The values range between 0 to 90 g/h/m^2^ while the relative humidity was from 0% to 100%.

The pH of the skin surface was measured with a pH meter PH 900 equipped with a planar probe head that combines the H+ ion sensitive electrode and the reference electrode in one rod. The probe was accurately calibrated with buffer pH 7 and pH 4 before the analyses. The Sebumeter SM 8 was used as probe and the quantity of sebum is related to the transparency of the tape analyzed by a photocell. The stratum corneum protein content (PC) was investigated through tape stripping using a D-Squame device [29]. This method involves the application of adhesive tape to skin and its subsequent removal to strip off a layer of the stratum corneum and the quantification was investigated with an infrared densitometry technique. Results are expressed as PC in μg/cm^2^.

#### 4.2.4. In Vivo Squalene Analysis

A procedure for the extraction and analysis of squalene in both peroxide and non-peroxide forms from volunteers with acne vulgaris and healthy volunteers as controls was developed. For the analyses, patients were not allowed to apply creams or make-up for at least 24 h before extraction to avoid any interference. The areas investigated were chin, cheek, forehead, and shoulder as control parameters of the same subjects as discussed in a previous work [17].

For the collection of sebum, D-Squame adhesive discs were used and a previously validated method was applied [17]. D-100-D-Squame Stripping Discs are a special and safe tape to collect human sebum without causing harm and pain to the subject. Briefly, the discs were applied with constant pressure for about 10 s. For each specific area, three samples were taken. Then, each individual strip was placed inside a 2 mL Eppendorf tube and then the squalene extraction was performed. Each strip was weighed before and after extraction to make a first quantification of the amount of sebum taken off.

##### Extraction Procedure

For squalene extraction, different methods were reported in the literature. Ayano Ishikawa and Shimizu N. extracted squalene using cotton swabs soaked in acetone and then extracted in chloroform [18,19]. Swarna Ekanayake Mudiyanselage, instead, used Sebutapes^®^ to collect sebum on volunteers soaked in 1 mL of ethanol solution 70% [20].

Once the tape strip was put into a 2 mL Eppendorf tube, 1.5 mL of HPLC-grade ethanol was added. The analyses were performed in a thermomixer at 25 °C, 800 rpm for 20′. Thereafter, the strips were removed, and the solution was centrifuged at 25 °C, 10,000 rpm for 10′. The supernatant was filtered through PTFE filters and the HPLC analyses were performed.

To confirm the method, an extraction was also carried out using a blank strip to verify that any interference was present. To compare the quantity of squalene in different parts of body of the same volunteers, the shoulder was chosen as control.

#### 4.2.5. High-Performance Liquid Chromatography (HPLC) Analyses

A scheme of HPLC parameters is reported in Table 3. 

Peak quantification and integration were performed with Thermo Scientific™ Chromeleon™ Chromatography Data System Software 7.3 (60919), Waltham, MA, USA.

##### HPLC Method Validation

For HPLC validation, ICH Q2 (R1) guidelines were followed [30]. In particular, the analyses focus on specificity, linearity, accuracy, intermediate precision, limit of detection (LOD) and limit of quantification (LOQ).

▪ For the measurement of precision, it is necessary to make repeated measurements over time and obtain similar results. For this purpose, triplicate analyses were performed on the same day, using a concentration of 90 ug/mL that corresponds to the midpoint of the calibration curve (intra-day assay). The same value was then used to perform triplicate analyses on two subsequent days (inter-day assay). Repeatability and intermediate precision results should not exceed a value of 5% [24].
▪ Eight different concentrations (5–200 μg/mL) were analyzed for three consecutive days to measure linearity. The analyses were performed in triplicate, obtaining the coefficients of determination R^2^.
▪ To assess the validity of the linear regression, the data obtained were subjected to statistical analysis with the test ANOVA one-way and significance level of α = 0.05 (95% confidence interval).
▪ The accuracy was determined by recovery of known amounts of squalene reference standard added to the samples at the beginning of the process. Briefly, 50, 70 and 100 μg/mL of standard squalene was added to sample. The percentage recovery of added squalene standard was calculated. Moreover, standard deviation (SD) and the relative standard deviation (RSD%) were determined.
▪ Limit of detection (*LOD*) and limit of quantification (*LOQ*) were based on standard deviation of the response and the slope. The detection limit (*LOD*) and the quantification limit (*LOQ*) may be expressed by the Formulae (1) and (2):

(1) LOD=3.3 σ/S

(2) LOQ=10 σ/S

where *σ* = the standard deviation of the response

*S* = the slope of the calibration curve

The linearity and the validity of the calibration curve, the analysis of variance (ANOVA) was used as a statistical test. A significant level of α = 0.05 (95% of confidence interval) was assessed, thus statistical difference is significative for *p* < 0.05.

#### 4.2.6. Standard Solutions and the Sample Preparation

A calibration standard stock solution of squalene was prepared by accurately weighing out 40 mg of squalene and dissolving it into 25 mL of acetonitrile. This stock solution was further filtered and diluted with the same solvent to obtain a range between 5–200 μg/mL of squalene.

To increase the peroxidation of squalene, the sample was placed under strongly oxidizing conditions. Therefore, it was decided to treat squalene with hydrogen peroxide. Hydrogen peroxide is an oxidizing agent, which induces the formation of peroxides; so, it can be used for the formation of peroxides. Two solutions were prepared: one of hydrogen peroxide (36 volumes, 10.8%) in mobile phase to verify that it would not interfere in the analysis, and one of squalene with hydrogen peroxide. The squalene solution with hydrogen peroxide was left in contact for 24 h. Then, the solution was placed in a solar simulator to increase the formation of peroxides.

A test tube containing 116.17 g of Sq and 3.2 g of H_2_O_2_ (36 volumes, 10.8%) was prepared. The moles of squalene in this solution are 0.283 mol; hydrogen peroxide at 36 volumes liberates 1.61 mol of oxygen. Being under conditions of excess oxygen liberation, all the oxidizable portions of squalene can undergo peroxidation.

For the preparation of peroxide squalene for HPLC injection, the previous method was performed. A solution of 1 mg/mL was prepared in acetonitrile and then diluted. Hydrogen peroxide was also injected to observe that there was no interference.

#### 4.2.7. In Vitro Peroxidation Analysis

The in vitro experiment to quantify the peroxide number (*NP*) of lipid was set up. The titration analysis involves the measurement of hydroperoxides expressed as milliequivalents of oxygen per kg of sample. Before *NP* determination, a sodium thiosulfate solution was standardized using KIO_3_ to obtain a normality of 0.002 N. Then, the oil was weighed (about 1 g) and placed in a flask. It is dissolved in a 3:2 mixture of glacial acetic acid and chloroform (25 mL). A generous amount of potassium iodide was added. The mixture was shaken and left in a dark room for 5 min. At this point, 75 mL of distilled water was added and 1 pipette of starch weld was used as redox indicator. The titration procedure was conducted using sodium thiosulfate in a 50 mL burette. The peroxide number (*NP*) was calculated using the following equation [29]:

(3) $$NP=\frac{V_{Na2S2O4\;\left(mL\right)*}\;N_{Na2S2O4\;}\left(\frac{eq}{L}\right)*1000}{ms\;(g)}=meq\;O_{2}/kg\;oil$$

*V_Na*2*S*2*O*4*_* = sodium thiosulfate volume

*N_Na*2*S*2*O*4*_* = calculated normality of sodium thiosulfate

*ms* = sample mass (*g*)

For the production of peroxides, the work of Ekanayake Mudiyanselage, S. was taken as reference [31] and modified. Fresh olive oil and peanut oil were used as positive controls. Squalane, the saturated form of squalene, was used as a negative control.

Briefly, all samples were exposed to solar radiation in solar simulator (Suntest ^®^ XLS+, Atlas Material Testing Solution, Filter: SOLAR ID65, 35 °C, 600 W/m^2^) to increase the peroxide number until the plateau was reached. To investigate the number of peroxides formed, the titration analyses were conducted at 18, 36, 54 and 72 h.

##### Statistical Analysis

A statistical analysis of the data collected from the “in vivo” studies was performed. The Spearman’s rank correlation test was used to evaluate the correlation between two variables [32]. It has a value between +1 and −1, where 1 is a total positive correlation and −1 is a total negative correlation. The correlation reflects the noisiness and direction of a relationship, but not the slope of that relationship, nor many aspects of nonlinear relationships. There is no general consensus on the classification of the relationship for different coefficients. In this work, the criteria reported in Table 4 were applied.

## 5. Conclusions

This work allows us to set a rapid and simultaneous test to collect squalene and its peroxide form in acne-prone skin compared with healthy volunteers. The tape stripping method provides a suitable approach for the collection of enough material for the quantification analysis without carrying pain and damage to the skin, increasing the compliance of subjects. Moreover, the simulation of peroxidation reaction induced by porphyrins using solar simulator has been demonstrated to be efficient to increase the number of peroxides dependent on the time exposure, and the quantification with sodium thiosulfate shows an efficient method for the analyses of NP.

A marker that distinguishes acne-prone skin in respect to healthy skin is a higher amount of squalene and the presence of its peroxide form in the acne skin. Squalene, therefore, could act as a lipid marker for this type of skin disease. Considering the therapies currently available for acne vulgaris along with their respective adverse effects, a new strategy could involve preventing and limiting squalene peroxidation by directly targeting porphyrins. This work represents a starting point to increase the knowledge about the methods to obtain and quantify some outcomes related to acne vulgaris. Surely, the following step could be the evaluation of microbiota composition that affects this skin disorder.

## Figures and Tables

**Table 1:** Results of regression analysis of data for the quantitation of squalene.

Parameters	
Regression equation	y = 0.7973x + 1.4809
Correlation equation	R^2^ = 0.9994
Range [ug/mL]	5–200
LOD [ug/mL]	9.72
LOQ [ug/mL]	29.45

**Table 2:** Skin parameters collected both from acne-prone and healthy skin, expressed as a mean ± S.D.

PARAMETERS	ACNE SKIN		HEALTHY SKIN		Variation Acneic vs. Healthy Skin (%)
TEWL (g/m^2^ h)	12.16 ± 2.56		9.85 ± 1.09		23.45
Protein content (µg/cm^2^)	STRIP 1	13.68 ± 1.92	STRIP 1	8.90 ± 2.60	53.71
	STRIP 2	11.82 ± 1.69	STRIP 2	8.70 ± 1.70	35.86
	STRIP 3	10.34 ± 1.95	STRIP 3	8.30 ± 1.35	24.58
pH	5.40 ± 0.22		5.18 ± 0.37		4.25
Sebum levels (µg/cm^2^)	172.2 ± 55.90		81.70 ± 36.35		110.77

**Table 3:** The operating conditions for squalene quantification in high-performance liquid chromatography (HPLC).

Parameters	
Column	CAPCELL PAK (Shiseido) C18 UG 120 Å 5 µm, 250 mm × 4.6 mm
Flow	1.5 mL/min
Column temperature	25 °C
Injection volume	50 μL
Wavelength	210 nm
Time course	10 min
Mobile phase	Ethanol/Acetonitrile 70:30 Isocratic elution
Retention time	4.096 min for squalene

**Table 4:** Criteria applied for the strength interpretation of Spearman’s rank correlation coefficient.

Coefficient Value		Strength Interpretation
+1	−1	Perfect positive or negative correlation
+0.9–0.7	−0.9–0.7	Very strong correlation
+0.6–0.4	−0.6–0.4	Strong correlation
+0.3	−0.3	Moderate correlation
+0.2	−0.2	Weak correlation
+0.1	−0.1	Negligible correlation
0	0	No correlation

## Data Availability

Data is contained within the article and supplementary material.

## Supplementary Materials

The following supporting information can be downloaded at: https://www.mdpi.com/article/10.3390/ph16121704/s1, Figure S1. Squalene calibration curve; Table S1. Linear range investigated over three days; Table S2. Analysis of variance (ANOVA) for linearity. Analysis of variance is used to determine whether multiple groups may be significantly different from each other in some way or, conversely, are homogeneous. in the between-group variance, results taken on 3 different days were considered, while in the within-group variance, data obtained each day were taken and compared for homogeneity; Table S3. Precision of the validation method; Table S4. Experimental values obtained in the recovery test for squalene.
