# Supplementary material for: Squalene Peroxidation and Biophysical Parameters in Acne-Prone Skin: A Pilot “In Vivo” Study
Source: Pharmaceuticals (Basel). 2023 Dec 8;16(12):1704. doi: 10.3390/ph16121704 (PMC10748031; doi:10.3390/ph16121704)
Supplement: Supplementary file 1 [file pharmaceuticals-16-01704-s001.zip › pharmaceuticals-2732670-supplementary.pdf]

Supplementary material

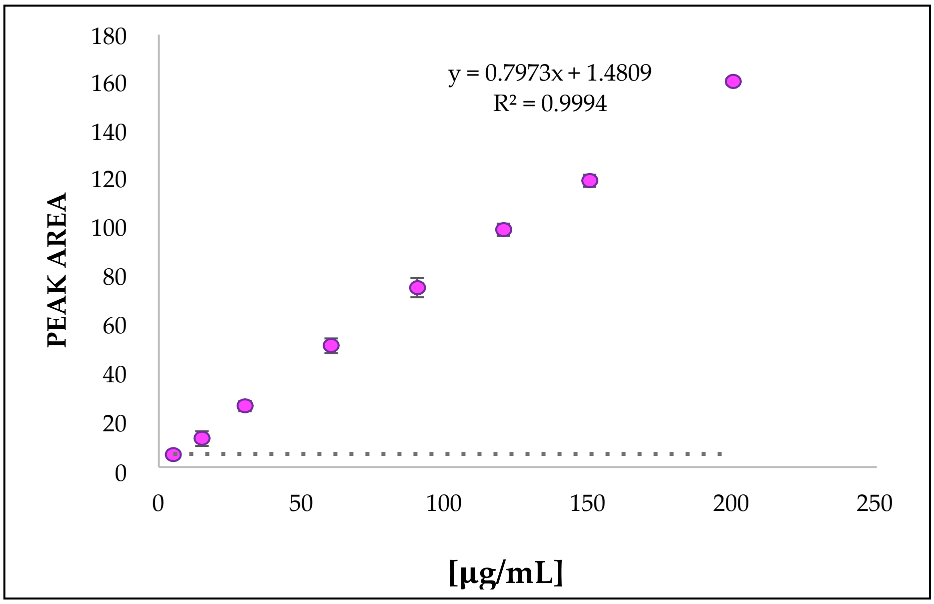

Figure S1. Squalene calibration curve

Table S1. Linear range investigated over three days.

		day 1	
replicate	slope	y-intercept	R^2
1	0.7957	2.8846	0.9970
2	0.8131	-1.6433	0.9994
3	0.7867	3.4519	0.9990
		day 2	
replicate	slope	y-intercept	R^2
1	0.790	0.3679	0.9950
2	0.811	-2.0127	0.9996
3	0.7758	3.0761	0.9948
		day 3	
replicate	slope	y-intercept	R^2
1	0.7831	3.4587	0.9910
2	0.8144	1.2348	0.9982
3	0.8121	1.7469	0.9981

Table S2. Analysis of variance (ANOVA) for linearity. Analysis of variance is used to determine whether multiple groups may be significantly different from each other in some way or, conversely, are homogeneous. in the between-group variance, results taken on 3 different days were considered, while in the within-group variance, data obtained each day were taken and compared for homogeneity.

Source of variation	SS	DF	MS	F calculated	p-value*	F tabulated
Between groups	24	2	12	0.0040	0.996	3.467
Within groups	64215	21	3058			
Total	64240	23				

Legend: SS-Sum of squares; DF-degrees of freedom; MS-Mean square

Table S3. Precision of the validation method

Validation parameters		
Precision intra-assay (Repeatability) (n=6 determinations)		RSD% 1.44
Theoretical concentration (µg/mL).	95	
Concentration obtained (µg/mL)		95.77
Precision inter-assay (Repeatability) (n=6 determinations)		RSD% 1.83
Theoretical concentration (µg/mL).		95
Concentration obtained (µg/mL)		94.67

Table S4. Experimental values obtained in the recovery test for squalene

[sample] ug/mL	[standard added] ug/mL	% recover ymean	RSD%
30	50	106.51	1.74
30	70	99.10	0.83
30	100	101.15	1.85
